# Epileptic seizures detection and the analysis of optimal seizure prediction horizon based on frequency and phase analysis

**DOI:** 10.3389/fnins.2023.1191683

**Published:** 2023-05-16

**Authors:** Ximiao Jiang, Xiaotong Liu, Youjun Liu, Qingyun Wang, Bao Li, Liyuan Zhang

**Affiliations:** ^1^Department of Biomedical Engineering, Faculty of Environment and Life, Beijing University of Technology, Beijing, China; ^2^Department of Dynamics and Control, Beihang University, Beijing, China

**Keywords:** electroencephalogram (EEG), phase-amplitude coupling (PAC), frequency-domain analysis, seizure prediction horizon (SPH), machine learning

## Abstract

Changes in the frequency composition of the human electroencephalogram are associated with the transitions to epileptic seizures. Cross-frequency coupling (CFC) is a measure of neural oscillations in different frequency bands and brain areas, and specifically phase–amplitude coupling (PAC), a form of CFC, can be used to characterize these dynamic transitions. In this study, we propose a method for seizure detection and prediction based on frequency domain analysis and PAC combined with machine learning. We analyzed two databases, the Siena Scalp EEG database and the CHB-MIT database, and used the frequency features and modulation index (MI) for time-dependent quantification. The extracted features were fed to a random forest classifier for classification and prediction. The seizure prediction horizon (SPH) was also analyzed based on the highest-performing band to maximize the time for intervention and treatment while ensuring the accuracy of the prediction. Under comprehensive consideration, the results demonstrate that better performance could be achieved at an interval length of 5 min with an average accuracy of 85.71% and 95.87% for the Siena Scalp EEG database and the CHB-MIT database, respectively. As for the adult database, the combination of PAC analysis and classification can be of significant help for seizure detection and prediction. It suggests that the rarely used SPH also has a major impact on seizure detection and prediction and further explorations for the application of PAC are needed.

## 1. Introduction

Epilepsy is a chronic brain disorder, characterized by recurrent seizures. The reason for the pathological dynamics is the abnormally synchronous discharge of groups of, in particular, cortical neurons (Tsipouras, 2019). Seizure onset can lead to loss of consciousness, disorders of mood, and, in extreme cases, even death of the patients (Yang et al., 2021). It affects nearly 50 million people worldwide (Acharya et al., 2017; World Health Organization Epilepsy, 2023). Seizures can be treated through drug treatment, surgical intervention, and neuromodulation (He et al., 2021; Mueller et al., 2022). However, in the process of treatment, inconsistent availability of clinical data, the complexity of epilepsy etiology, and the lack of standard diagnostic procedures often make diagnosis and follow-up treatment difficult. Thus, exploring effective methods to detect and predict seizure onset is an important Research Topic.

The electroencephalogram (EEG) measures the electrical activity of the brain and is thus an important examination tool for the clinical diagnosis of neurological disorders including epilepsy and Alzheimer's disease (Cho et al., 2017; Yu et al., 2020). According to the collection method, there are two common types of EEG recordings, namely, scalp electroencephalography (scalp EEG) and intracranial electroencephalography (iEEG) (Jayakar et al., 2016). In humans, oscillatory brain activity occurs in a variety of frequency bands reflecting electrophysiological signals generated by large ensembles of synchronized neuronal firing (Jensen and Colgin, 2007). Specifically, the amplitude of high-frequency oscillation has been suggested as a biomarker of the seizure onset area (Charupanit et al., 2020). In clinical practice, the diagnosis is typically based on a patient's clinical representation and available multimodal data. However, this has some disadvantages, particularly being time-consuming (Vidyaratne and Iftekharuddin, 2017; Duan et al., 2022). Concerning the EEG, in addition to contributions from neural activity, the signals contain interfering signals from other sources, which may make the diagnosis difficult. There is a need for reliable algorithms, specifically for automatic seizure onset detection as recorded in the EEG.

In recent years, researchers have designed various methods to extract various features from EEG recordings. There are three main analysis methods, namely time domain analysis, frequency domain analysis, and time–frequency domain analysis. In terms of frequency domain analysis, researchers have extracted various features, including mean frequency and root mean square, and achieved an excellent result on seizure detection. Cross-frequency coupling (CFC) is a method to dynamically measure interactions of neural oscillations in different frequency bands and between brain areas. CFC also appears to detect neural correlates of various cognitive states (Liu et al., 2018). Three types of algorithms, namely phase–amplitude coupling (PAC), phase–phase coupling (PPC), and amplitude–amplitude coupling (AAC), are common methods for CFC analysis (Munia and Aviyente, 2021). Among them, PAC, which quantifies the interplay between the amplitude of high-frequency oscillations and the phase of low-frequency oscillations, has recently become a topic of interest (Munia and Aviyente, 2019). In the context of epilepsy, it was shown that interictal PAC is helpful for the localization of the epileptogenic zone (Motoi et al., 2018; Ma et al., 2021). Although most studies emphasized PAC analysis for seizure onset zone (SOZ) detection, few studies have also applied it to the (temporal) detection and prediction of seizure onset (Edakawa et al., 2016; Grigorovsky et al., 2020; Yamamoto et al., 2021). Due to the non-linearity and non-stationarity of EEG signals, the synchronization process of epilepsy is also discussed to analyze the mechanism as well as the complex underlying dynamics of seizure (Fan and Chou, 2019). For quantitative analysis, functional brain networks and graph theory have provided opportunities to understand the complex mechanism changes (Yu et al., 2018; Akbarian and Erfanian, 2020; Fallahi et al., 2021; Liu et al., 2021). The network metrics including the efficiency, clustering, small worlds, and modular organizations are the meaningful information to extract the topological properties of the brain network.

Recently, machine learning with powerful computing ability has made available algorithms to potentially improve classical data analysis. A variety of machine learning models have been proposed for classification. Common classification algorithms include the support vector machine (Hussain, 2018), decision trees, K-nearest neighbor (Jukic et al., 2020), and random forest (Sun Q. et al., 2021). In Sun Q. et al. (2021), the authors combined the random forest algorithm with time domain and non-linear characteristics for seizure detection and were able to obtain a high accuracy of state classification. Similar methods based on the random forest algorithm have been applied to differentiate between types of seizures and achieved a good performance (Basri and Arif, 2021). A method combining machine learning and functional brain networks has been adopted by researchers in more and more fields. Yu et al. (2019) applied it to automatically identify acupuncture manipulations and with the support vector machine algorithm, the highest accuracy can be obtained. With the improvement and optimization of algorithms and models, deep learning has also gradually been applied to the study of epilepsy. Convolutional Neural Network (CNN) has stood out and was applied in many research in terms of image recognition (Ryu et al., 2021; Wang et al., 2022). Compared with the conventional CNN, Graph Convolutional Network (GCN) can preserve rich marginal features having the advantage of explaining the connective relationships between features (Chen et al., 2021; Jia et al., 2022; Li et al., 2022). The deep learning method acquires abundant EEG data. However, rare public datasets can provide such an amount of EEG data which is a wicked problem.

In the context of seizure detection and prediction, the main goal is to classify the interictal stage and the preictal stage (Snyder et al., 2008; Yang et al., 2021). To achieve that, the seizure prediction horizon (SPH) and the seizure occurrence period (SOP) were suggested (Maiwald et al., 2004). The SOP is a time period when a seizure is predicted to occur and the SPH is the interval from the alarm to the beginning of the SOP. A correct prediction is achieved when a seizure onset occurs after the SPH and within the SOP. Recently, studies have addressed the problem of the length of SPH and SOP. Wang et al. compared the prediction effect of SOP between 30 and 60 min with the SPH of 5 min achieving an excellent performance (Wang et al., 2022). Moreover, Aarabi et al. conducted prediction experiments on iEEG data with an SOP of 30 and 50 min and an SPH of 10 s (Aarabi and He, 2017). Additionally, Zhang et al. acquired a high sensitivity by setting SPH to zero. In contrast, few studies emphasized the length of SPH which was also called the intervention time (Wang et al., 2022). In clinical practice, it was still important to find an appropriate SPH to leave enough time for providing effective intervention.

In this study, we propose a method for epileptic seizure onset detection and for the classification of preictal and interictal states. Frequency domain analysis is performed on two databases, the Siena Scalp EEG database and the CHB-MIT database. Based on the single-channel analysis, the length of SPH is adjusted to find the optimal SPH for potential treatment.

## 2. Material and methods

**Figure 1:**
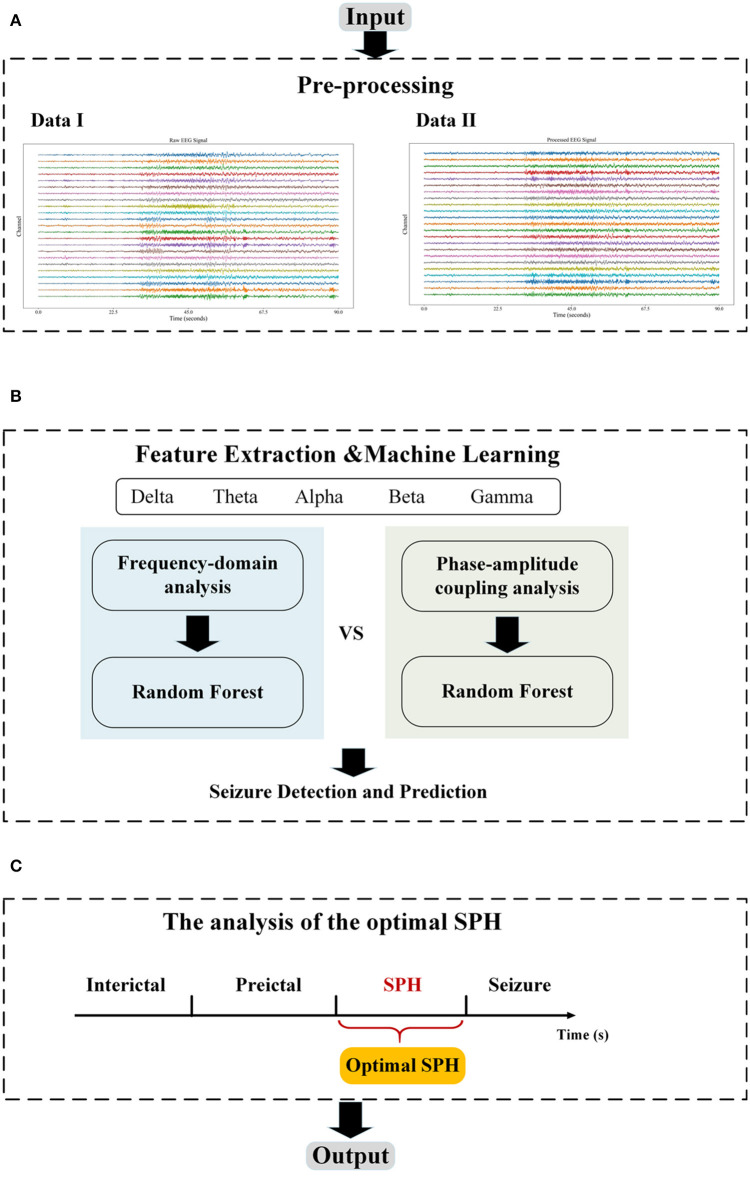
The flowchart. **(A)** The pre-processing module includes filtering and Independent Components Analysis (ICA); **(B)** The feature extraction module includes frequency domain and PAC, and the machine learning module performs the classification; and **(C)** The optimization module adjusts the length of SPH to find the optimal SPH. The data I in **(A)** are the raw EEG data from chb01 in the CHB-MIT database and the Data II are the output of the pre-processing module.

This section describes the database used for the experiment and the method, which can be categorized into three major parts: first, the EEG signal is preprocessed; second, features including single-channel PAC, peak frequency, and median frequency are extracted; and third, the random forest classifier is applied for classification. The flowchart of the proposed method in this study is illustrated in Figure 1.

### 2.1. Database

Two databases were used in this study: the Siena Scalp EEG database and the CHB-MIT database.

The Siena Scalp EEG database (https://www.physionet.org/content/siena-scalp-eeg/1.0.0/) collected by the Unit of Neurology and Neurophysiology at the University of Siena (Detti et al., 2020). It consists of scalp EEG recordings from 14 patients including nine men (ages 36–71) and five women (ages 20–58). The recordings were captured with a sampling rate of 512 Hz, with electrodes arranged on the international 10–20 system. In total, this database has a component of 47 seizures on about 128 recording hours. In the study, we used 29 channels (“Fp1”, “F3”, “C3”, “P3”, “O1”, “F7”, “T3”, “T5”, “Fc1”, “Fc5”, “Cp1”, “Cp5”, “F9”, “Fz”, “Cz”, “Pz”, “F4”, “C4”, “P4”, “O2”, “F8”, “T4”, “T6”, “Fc2”, “Fc6”, “Cp2”, “Cp6”, “F10”, and “Fp2”). Table 1 reports the details of the data.

**Table 1:** Data of the patients in the Siena Scalp EEG database.

**Patient**	**Gender**	**Age (years)**	**EEG Chan**.	**Seiz**.
1	M	46	29	2
3	M	54	29	2
5	F	51	29	3
6	M	36	29	5
7	F	20	29	1
9	F	27	29	3
11	F	58	29	1
12	M	71	29	4
13	F	34	29	3
14	M	49	29	4
16	F	41	29	2
17	M	42	29	2

EEG Chan., the number of EEG channels; Seiz., the number of seizures; F, Female; M, Male. For comparison, the patient numbers here are marked in the original database without modification.

The second database is the CHB-MIT database (https://www.physionet.org/content/chbmit/1.0.0/) which contains the widely used scalp EEG recordings from 23 patients at Children's Hospital Boston (Shoeb, 2009; Truong et al., 2018; Yang et al., 2021). Among them, chb21 was obtained 1.5 years after case chb01, from the same female subject, and chb24 with incomplete information was added to the database later. The records were captured at a rate of 256 samples per second sampling by 16-bit resolution using the International 10–20 Electrode Position System. A total of 983 h of consecutive EEG recordings and 198 seizures are available in the database. In most cases, files contain only 1 h of digitized EEG signal, although files belonging to case chb10 are 2 h, and files belonging to cases chb04, chb06, chb07, chb09, and chb23 are 4 h. In this study, we used 22 channels (“FP1-F7”, “F7-T7”, “T7-P7”, “P7-O1”, “FP1-F3”, “F3-C3”, “C3-P3”, “P3-O1”, “FP2-F4”, “F4-C4”, “C4-P4”, “P4-O2”, “FP2-F8”, “F8-T8”, “T8-P8”, “P8-O2”, “FZ-CZ”, “CZ-PZ”, “P7-T7”, “T7-FT9”, “FT9-FT10”, and “FT10-T8”) contained in most records. Table 2 reports the details of the data.

**Table 2:** Data of the patients in the CHB-MIT database.

**Patient**	**Gender**	**Age (years)**	**EEG Chan**.	**Seiz**.
1	F	11	23	7
2	M	11	23	3
3	F	14	23	7
4	M	22	23	4
5	F	7	23	5
6	F	1.5	23	10
7	F	14.5	23	3
8	M	3.5	23	5
9	F	10	23	4
10	M	3	23	7
11	F	12	23	3
12	F	2	23	40
13	F	3	23	12
14	F	9	23	8
15	M	16	31	20
16	F	7	28	10
17	F	12	28	3
18	F	18	22	6
19	F	19	28	3
20	F	6	28	8
21	F	13	28	4
22	F	9	28	3
23	F	6	23	7
24	-	-	23	16

EEG Chan., the number of EEG channels; Seiz., the number of seizures; F, Female; M, Male.

### 2.2. Pre-processing

To obtain valid features of the signal, pre-processing including filtering and ICA is essential. By appropriate filtering, the noise in EEG data can be effectively reduced. In other words, in a given frequency band, EEG signals can be filtered to improve the corresponding signal-to-noise ratio. The raw EEG signals were contaminated by power line contributions at 60 Hz and 50 Hz for the CHB-MIT and the Siena, respectively. Therefore, a notch filter was utilized to remove this power-line interference. The filtered data were processed by ICA to remove physiological artifacts, e.g., eye movements and other muscular noise. For further analysis, the preprocessed EEG data were decomposed into the classical EEG frequency bands using a fifth-order Butterworth bandpass filter.

**Figure 2:**
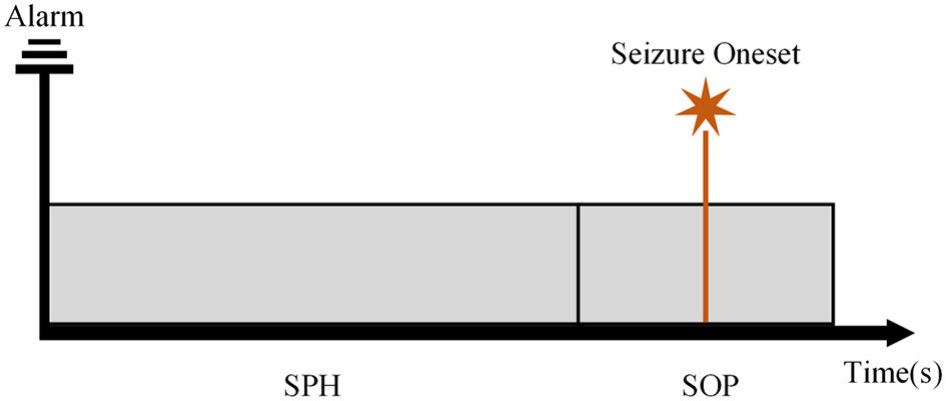
The schematic diagram of the seizure prediction horizon (SPH) and the seizure occurrence period (SOP).

We followed the definition of SPH and the SOP mentioned in Maiwald et al. (2004), Zhang and Parhi (2016), and Shokouh Alaei et al. (2019) as illustrated in Figure 2 for state division. The seizure onset may not occur immediately and exactly after the SPH, which indicates the uncertainty of the prediction. To better achieve the prediction, we assumed that the seizure onset was followed by the SPH in our experiments. For an effective and practical prediction, the SPH should not be too long. At the same time, from a clinical perspective, the SPH ought not to be too close to the seizure onset to allow for an intervention of patients. In the case of seizure clusters, we focus on the leading seizure. Thus, when a second seizure starts soon after the previous seizure, we considered them as only one seizure.

**Figure 3:**
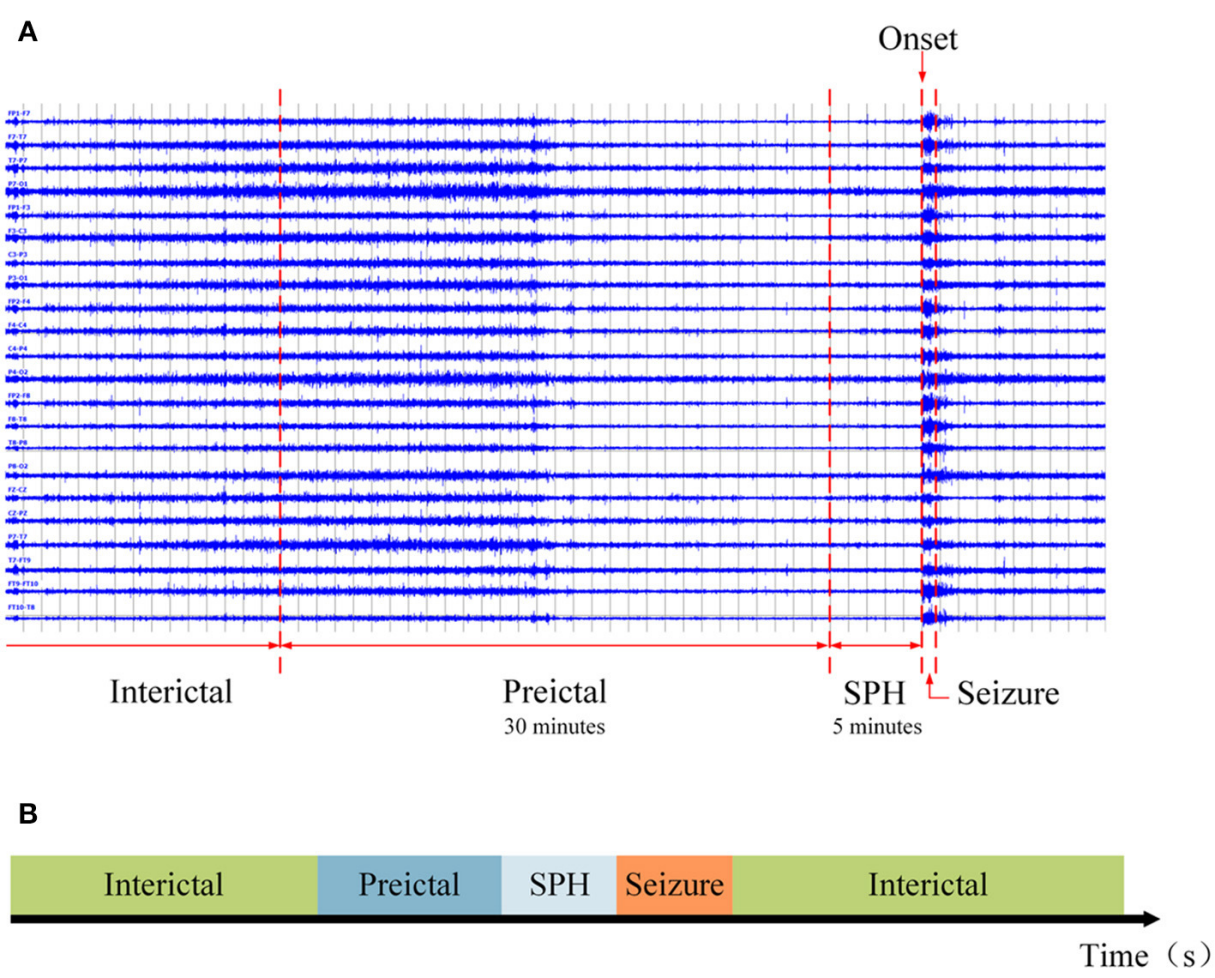
The ictal, SPH, preictal, and interictal states. **(A)** An example of a segmented EEG signal. The rightmost line is a landmark of the epileptic seizure onset. The SPH is set 5 min before seizure onset, the preictal state is 30 min earlier than SPH, and the interictal state follows the preictal state. **(B)** A schematic diagram of the segmentation.

According to Truong et al. (2018) and Sun B. et al. (2021), the preictal length was set to 30 min. Referring to Ryu et al. (2021), the SPH existed before the ictal state, and the time after the preictal state was assumed to be 5 min. At the same time, the rest of the recording was defined as an interictal state. The final partition of each state is shown in Figure 3. PN00 and PN10 in the Siena Scalp EEG database have insufficient preictal and interictal data. After removal, 12 subjects from this database were used.

For a specified EEG sequence, an EEG window length of 30-s with a slide step of 15-s was used to obtain 30-s segments of EEG signals (Truong et al., 2018).

### 2.3. Feature extraction

The single-channel PAC was calculated. For comparison, two common frequency domain features, namely, peak frequency and median frequency, were also extracted (Sánchez-Hernández et al., 2022).

Traditionally, PAC can be calculated as follows (Dupré la Tour et al., 2017). First, a bandpass filter is performed to decompose the EEG signal *x*(*t*) on each channel into low frequency *f*_*x*_ and high frequency *f*_*y*_, and the range is divided into delta (0.5–4 Hz), theta (4–8 Hz), alpha (8–13 Hz), beta (13–30 Hz), and gamma (30–80 Hz), which are the commonly used frequency bands for human EEG (Liu et al., 2021). Second, the Hilbert transform is applied to obtain the phase sequence Φ_*x*_ of a low-frequency band and the amplitude sequence *a*_*y*_ of a high-frequency band. Third, a metric is used to quantify the correlation between Φ_*x*_ and *a*_*y*_. In this study, the modulation index (MI) is chosen, which is robust against noise and short data epochs and overall the commonly used measurement method (Hulsemann et al., 2019; Munia and Aviyente, 2019; Liu et al., 2021; Ma et al., 2021).

To calculate the value of MI, we refer to Tort et al. (2008), in which 18 bins of 20° each are used (−180°-180°). The average amplitude of the high-frequency component is computed and normalized as follows:


(1) $$\begin{array}{l} P\left(j\right)=\frac{{\bar{f}}_{y}\left(j\right)}{\overset{N}{\underset{i=1}{\sum }}{\bar{f}}_{y\left(i\right)}} \end{array}$$

where ${\bar{f}}_{y}\left(j\right)$ is the average of *a*_*y*_ within each bin, *N* is the total number of bins, and the range of *j* is [1, *N*] (Fujita et al., 2022). Subsequently, the Shannon entropy is calculated by the following formula:


(2) $$\begin{array}{l} H\left(P\right)=-\overset{N}{\underset{j=1}{\sum }}P\left(j\right)logP\left(j\right) \end{array}$$

Here, *P* is the vector of the normalized averaged amplitude in each bin and *N* is the total number of bins. The Shannon entropy depends on the number of bins and so does the MI. According to Tort et al. (2008) and Hulsemann et al. (2019), 18 bins were employed.

PAC is significantly associated with the deviation from the uniform distribution. The Kullback–Leibler distance, a measure for the disparity of the distributions, is calculated by the following formula:


(3) $$\begin{array}{l} KL\left(U,X\right)=\log N-H\left(P\right) \end{array}$$

where *U* is the uniform distribution, *X* is the distribution of the data, *N* is the total number of bins, and log*N* is the maximum entropy value. The final MI is computed as follows:


(4) $$\begin{array}{l} MI=\frac{KL\left(U,\;X\right)}{\log N} \end{array}$$

where *KL*(*U, X*) is the Kullback–Leibler distance according to Eq. 3 and *N* is the total number of bins.

We used the Welch function to obtain the signal power spectrum for each band, and the peak frequency and median frequency were calculated to characterize the highest peak in the power spectral density (Sánchez-Hernández et al., 2022).

### 2.4. Classification

A classical machine learning algorithm was employed for the classification based on the extracted features. As an ensemble learning algorithm, the random forest classifier stands out among traditional classifiers (Basri and Arif, 2021). It is based on ensemble decision trees trained by the bagging method. For an input sample, *M* trees will have *M* classification results. The algorithm then integrates all the classification voting results and designates the category with the most votes as the final output.

For the input data *D*, max–min normalization was used according to the given formula:


$$\begin{array}{lll} D_{scaled} & = & \frac{D-D.\min\left(axis=0\right)}{D.\max\left(axis=0\right)-D.\min\left(axis=0\right)}\;*\;\left(max-min\right) \end{array}$$

(5) $$\begin{array}{ll} + & \min \end{array}$$

Where max and min are the maximum and minimum values of the given mapping range. In our experiments, the mapping range was set to be (−1, 1).

As for the parameters adjustment, three parameters, namely, estimator, min-sample-split, and max-depth, were selected, and the grid search method was applied to find the best parameter value.

For the division of the data into the training and testing sets, the k-fold cross-validation method was employed for k=10 (Sameer and Gupta, 2020). Based on the seizures, the extracted features were randomly divided into 10 equal parts, nine of which were used for training and one for testing.

### 2.5. Statistical analysis

In this study, the analysis was conducted on a workstation with the Python 3.8.8 configuration as shown in Table 3. The overall goal was to classify the interictal and preictal states and to predict the ictal state. To evaluate the performance of the model, four evaluation metrics were calculated, namely, accuracy, precision, recall, and F-1 score, given as follows:


(6) $$\begin{array}{lll} Accuracy & = & \frac{TP+TN}{TP+TN+FP+FN} \end{array}$$

(7) $$\begin{array}{lll} Precision & = & \frac{TP}{TP+FP} \end{array}$$

(8) $$\begin{array}{lll} Recall & = & \frac{TP}{TP+FN} \end{array}$$

(9) $$\begin{array}{lll} F\text{-}1\;score & = & \frac{2^{*}Precision^{*}Recall}{Precision+Recall} \end{array}$$

The true positive (TP) is the number of segments that are correctly classified as preictal. The true negative (TN) is the number of segments that are correctly identified as interictal. The false positive (FP) represents the number of segments that are incorrectly classified as preictal, and the false negative (FN) represents the segments that are incorrectly recognized as interictal.

**Table 3:** Workstation configuration.

**Library**	**Version**
Numpy	1.18.5
Scipy	1.4.1
Scikit-learn	0.24.1
MNE	0.23.0

## 3. Results

### 3.1. The performance of seizure detection and prediction based on frequency domain analysis

**Figure 4:**
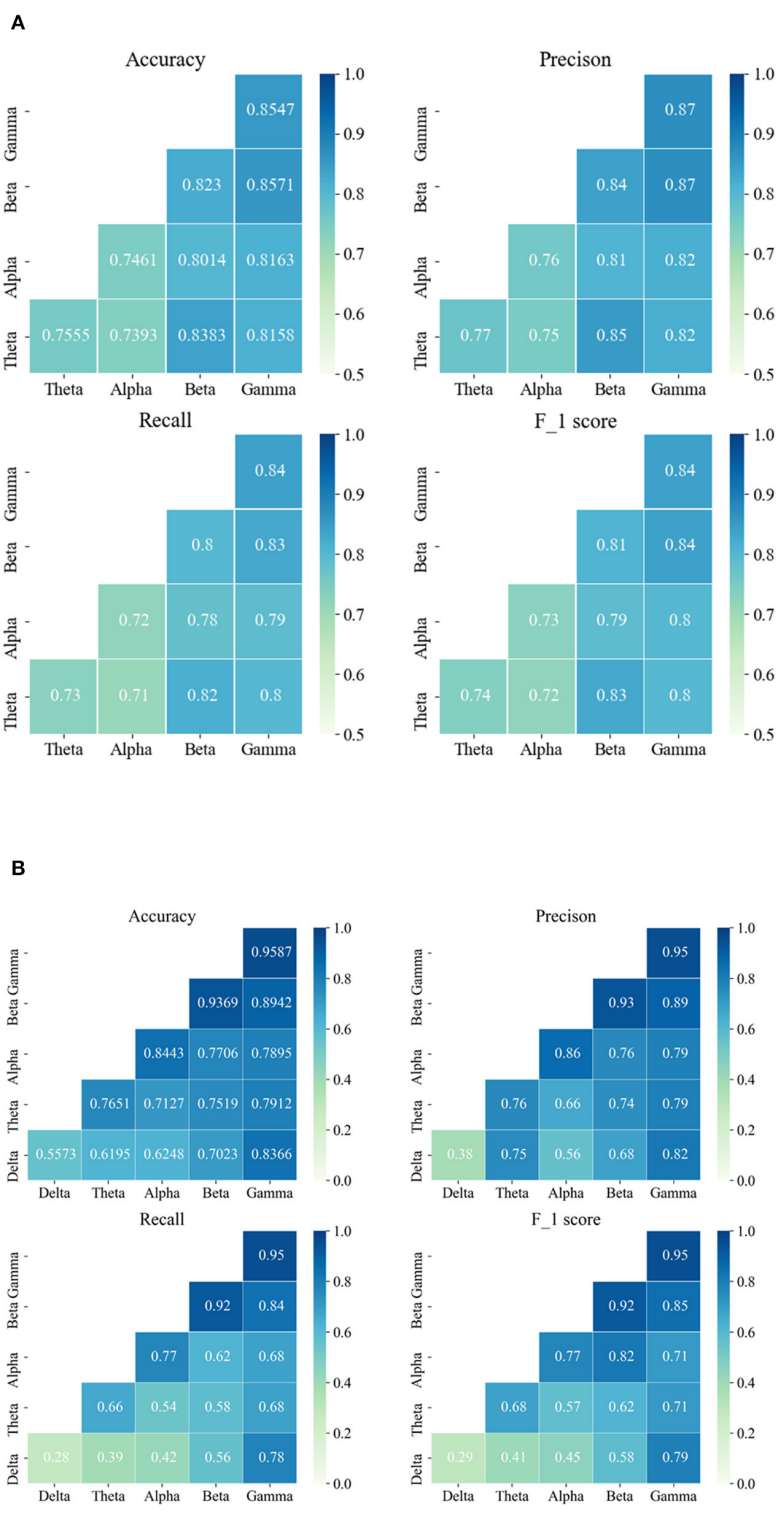
The seizure detection and prediction average results are based on the single-channel frequency domain analysis and PAC analysis, respectively. **(A)** The average results of the Siena Scalp EEG database are based on a single channel. **(B)** The average results of the CHB-MIT database are based on the single channel. The data on the diagonal represent the results of frequency domain analysis and the data on the lower triangle represent the results of PAC analysis.

The average results of a single-channel frequency domain analysis based on the different sub-bands are provided in Figure 4. From the results of both databases, it can be seen that a better performance occurs at high frequencies, i.e., either the beta or gamma frequency band. For the Siena Scalp EEG database, the best performance was obtained in the gamma band, followed by the beta band. For the CHB-MIT database, an accuracy of 95.87% was achieved with the gamma band. Similarly, a high accuracy also can be obtained with the beta band. By comparing the results of different frequency bands, the performance weakens from the high- to low-frequency band, which suggests that the high-frequency band has more valid information for classification. Particularly in the CHB-MIT database, we observe that all evaluation metrics have improved a lot. For patients in the Siena Scalp EEG database, the EEG data came pre-processed by high-pass filtering at either 1.6 Hz or 5.3 Hz. We, therefore, excluded the delta band and evaluated the other four bands.

### 3.2. The effect of seizure detection and prediction based on phase–amplitude coupling analysis

For further exploration, the MI was computed and the average PAC results are shown in Figure 4. Based on the results, the beta-gamma leading AC has the best performance on both databases. To be specific, the highest average accuracy of 85.71% was obtained on the Siena EEG database, while an average accuracy of 89.42% was achieved on the CHB-MIT database. Apart from that, it can be found that beta-theta leading PAC also had a good performance on the Siena EEG database. For the Siena Scalp EEG database, the average accuracy was enhanced by 4.13% from 81.58% to 85.71%; for the CHB-MIT database, the average accuracy improved by 5.76% from 83.66% to 89.42%.

**Figure 5:**
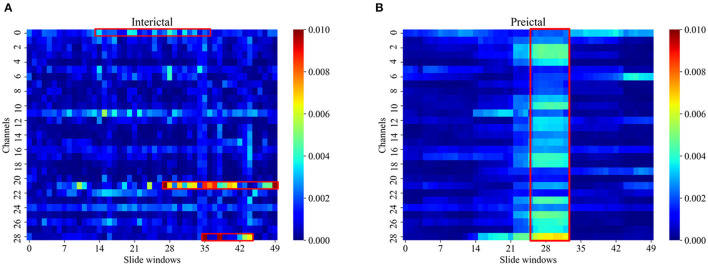
The MI pseudo-color graph of interictal **(A)** and preictal **(B)**. The MI was computed in 30 s windows shifted by 15 s for all electrode channels of patient 07 in the Siena EEG database. The usually significant channels and time points of change were labeled by red rectangle boxed.

Figure 5 illustrates the MI pseudo-color graph of interictal and preictal for all electrode channels with the 30-s slide windows moved. PAC presented a different characteristic in the interictal and preictal state. As shown in Figure 5, the interictal PAC was rare and weak, while the preictal PAC bursts rhythmically during some periods of time, which were indicated by red rectangle boxes. Also, PAC can occur on different channels at different times, indicating that the PAC varied with time.

Comparing the results of the single-channel frequency domain analysis with the PAC, we can find some nuances in both databases. For the Siena EEG database, the adult database, the best result was obtained by applying the PAC method; for the CHB-MIT database, the child database, the single gamma frequency band achieved the best performance. More interestingly, for the CHB-MIT database, the delta band with other high-frequency bands and PAC can improve the results compared with the single delta band.

### 3.3. The results of seizure detection and prediction based on the analysis of optimal SPH

The above results are based on the SPH of 5 min (Truong et al., 2018; Ryu et al., 2021; Wang et al., 2022). In this section, we adjust the length of SPH to between 10 and 15 min, respectively, to analyze the influence on seizure detection and prediction caused by the length of SPH. Based on the above results, the following analysis is conducted on the gamma band (frequency domain analysis) and the beta-gamma PAC, the optimal frequency band, for the CHB-MIT database and the Siena EEG database, respectively.

Supplementary Tables 1, 2 show the accuracy, precision, recall, and F-1 score for each patient from two databases according to the length of SPH. For each patient, there are three major outcomes. First, for most patients, a higher accuracy can be achieved when the SPH of 5 min was used. Second, some patients had better accuracy with the SPH of 10 or 15 min. Third, there was no difference for different lengths of SPH. For the first two cases, we selected patient 01 and patient 05 from the Siena EEG database, for example. The former obtained an accuracy of 87.12% and 79.53% at an SPH of 5 and 15 min, respectively. The latter obtained an accuracy of 81.47% and 88.73% at an SPH of 5 and 10 min, respectively. In the third case, for some patients from the CHB-MIT database, high accuracy can be obtained at these three interval lengths. What needed to be noticed was that some patients (e.g., 11 from the CHB-MIT database) have very few valid seizures considering the definition of the SOP and SPH displayed in Figure 2 and results do not vary with the interval length. There was no result with an SPH of 15 min due to the lack of a preictal state in the data.

**Figure 6:**
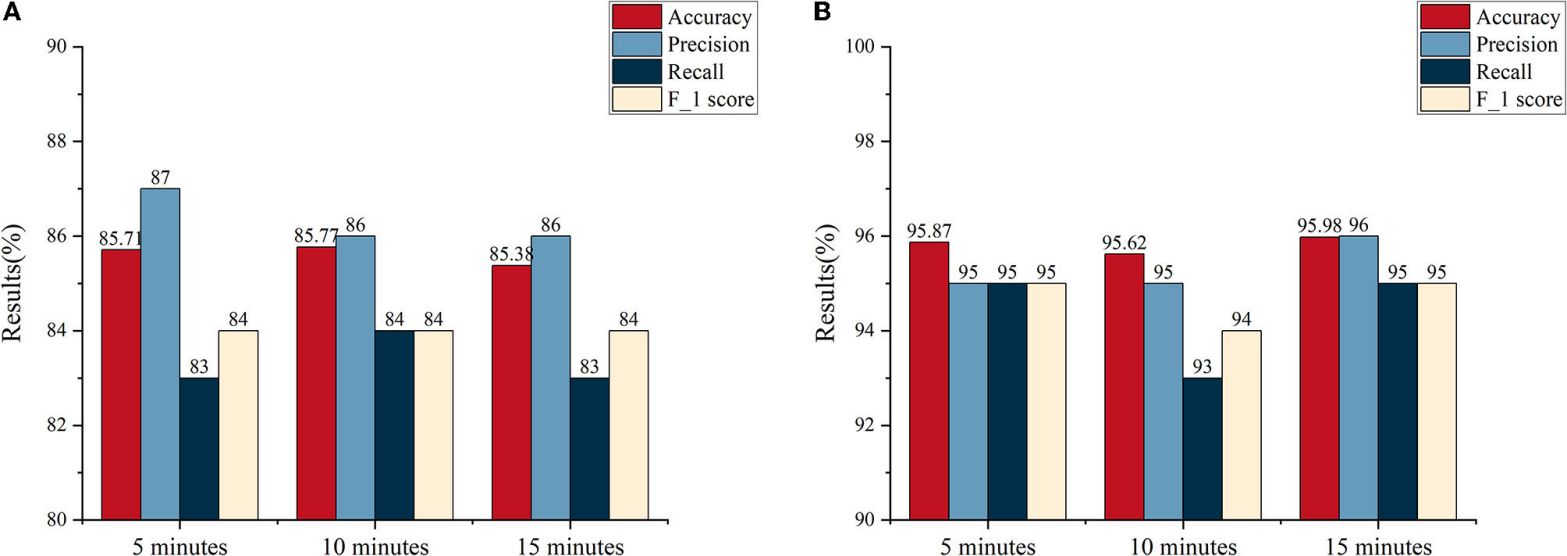
The average results of interval lengths of 5, 10, and 15 min, respectively. **(A)** The average results of the Siena Scalp EEG database. **(B)** The average results of the CHB-MIT database.

An average accuracy, precision, recall, and F-1 score are illustrated in Figure 6. Combining the results of the two databases, comprehensively, we find that the best performance was obtained with an SPH of 5 min. Although, the accuracy at the SPH of 5 min does not have a predominant advantage on both databases. There was, in terms of numerical results, a slight difference among the SPH of 5, 10, and 15 min. Supplementary Figures 1, 2 plot the results of the Kruskal–Wallis test between 5 min, 10 min, and 15 min SPH from the two databases. The *p*-values of the two databases are all more than 0.05. It suggests that at a certain range, the change of SPH has no significant effect on the accuracy of detection. Therefore, it is necessary to take the time expense to cure the patients and the feeling of the patients into consideration. If the length of the SPH is extended, it can increase the psychological stresses of patients. With a similar accuracy, thus, the SPH of 5 min can detect and predict the seizure faster and more efficiently.

## 4. Discussion and conclusion

In this study, frequency domain and PAC analysis are used to classify the interictal and the preictal states for seizure detection and prediction in EEG recordings from two cohorts of patients with epilepsy. The PAC classification analysis with the random forest classifier achieved better overall performance on the Siena Scalp EEG database for adults compared to the pediatric CHB-MIT database. In particular, the beta-gamma PAC stands out. The frequency domain analysis had the best performance on the pediatric CHB-MIT database. With both methods of feature extraction, the results improved from low- to high-frequency bands. In terms of the length of SPH, comprehensively, we found that the best overall performance was obtained with an SPH of 5 min, although some patients also had a good performance when the SPH was 10 or 15 min. Clinically, the highest accuracy does not necessarily mean practicality, that is the length of the warning time and the accuracy of the analysis need to be weighed against each other. Overall, it is found in this study that applying the SPH of 5 min can contribute to a better performance for seizure prediction, which has greater value for clinical prevention.

There is growing evidence that oscillatory activity in the brain plays a role in cognitive activities including sensory processing, feedback processing, and working memory (Jensen and Colgin, 2007). Scalp EEG recording of epilepsy was used in this study, which provides a general reflection of the activity of neurons on the scalp surface, important for clinical diagnosis, focal potentials, and postoperative review (Kobayashi et al., 2012; Tatum et al., 2018). High-frequency oscillation is considered to be a distinctive feature of the epileptogenic zone (Melani et al., 2013; Nariai et al., 2017). The acquisition of scalp EEG is more uncertain than that of iEEG. Artifacts from data preprocessing, muscle signals, and other factors can interfere with the analysis. Despite the above-described interference occurring during processing, there is a difference between the high-frequency activity caused by it and the pathological high-frequency rhythm produced by seizures (Kobayashi et al., 2004; Otsubo et al., 2007). Also, studies have highlighted that ictal slow waves are associated with ictal gamma rhythms. In spasms showing beta activity, the gamma rhythm is superimposed with it (Melani et al., 2013). This is a characteristic that is not present in non-pathological high-frequency rhythms. Consequently, we added cross-frequency coupling features to the frequency domain features to help predict seizures.

With unique coupling properties, CFC has been widely investigated in this context. Due to the distinctive and persistent PAC, researchers put much attention to its analysis during the seizure. However, the PAC of the interictal period and the preictal period are also of importance. Fujita et al. (2022) indicated that compared with healthy controls, epilepsy patients have abnormal PAC characteristics that can promote the discrimination between epileptic and normal. Amiri et al. (2016) found that the increased PAC is likely to be a sign of some fundamental abnormality in the interictal state. Ma et al. (2021) came to the conclusion that being paroxysmal, PAC of the interictal state and the preictal state can be used for accurate location of the epileptogenic zone. Also, the coupling of PAC can vary during the seizure. According to our results, there are distinct differences between the PAC of the interictal and the preictal state which is observed in Figure 5. It suggests that the PAC can help to classify them, particularly in the beta-gamma coupling band. Moreover, the proposed approach yields better results in the higher frequency bands. This is consistent with prior findings where the high-frequency range has a crucial role in cognitive function (Cho et al., 2017).

The incidence rate of epilepsy is particularly high in infancy and childhood. The characteristics of early infant EEG are various spatially distributed activities, rather than the more typical posterior rhythm in the mature EEG (Rosch et al., 2018). In addition, the electrographic symptoms of seizures in children are not as typical as those in adults. Lee and Lee (2013) indicated that, in terms of clinical features and interictal EEG, there were significant differences between patients who had temporal lobectomy in childhood and those having the operation during adulthood. Because the brain function of children is immature, it can easily be affected by adverse factors inside and outside the skull, potentially resulting in seizures. Most of the current studies have used data from the CHB-MIT database, which contains data from pediatric patients. Therefore, the Siena Scalp EEG database which is made up of adult data was added to our investigation to give a more comprehensive picture.

There are some limitations. First, our approach for seizure prediction is suitable only for EEG signals recorded continuously over a long period of time. The length of the data has a great influence on the final performance. Another limitation is that there are significant differences among different patients whose characteristics and dynamics of the peri-ictal states vary greatly (Yang et al., 2021). The proposed approach may thus not be suitable for all types of epileptic seizures. As displayed in Supplementary Tables 1, 2, it can be seen that, in terms of the length of SPH, the variation of the results is not uniform for each patient. A possible explanation for this is that they contain different types of seizures. Consequently, further work could consider the influence caused by the specific seizure type. Moreover, in the process of research, we found that the PAC has temporal and spatial differences. Since the two databases were public, however, the experiments were lack of patients' specific clinical information.

We compared the results of our work with previous studies in terms of SPH. To the best of our knowledge, there are only a few studies of SPH based on the CHB-MIT database. Table 4 gives the details including subjects, feature extraction method, classifier, SPH, and the four evaluation metrics based on the CHB-MIT database. As shown in Table 4, the method combining DWT and DenseNet-LSTM in Ryu et al. (2021) achieved an accuracy of 0.9328 and an F-1 score of 0.923. Hu et al. (2019) obtained an accuracy of 0.8625 with an SPH of 20 min. Compared to this, our method with an SPH of 5 min has a better performance.

**Table 4:** The comparison of seizure detection and prediction with other algorithms based on the CHB-MIT database.

**Authors**	**Subjects**	**Features**	**Classifier**	**SPH (minutes)**	**Acc**	**Pre**	**Recall**	**F-1**
Ryu et al. (2021)	24	DWT	DenseNet-LSTM	5	0.9328	-	-	0.923
Hu et al. (2019)	24	MAS	CNN	20	0.8625	-	-	-
This work	24	Peak frequency and median frequency	Random forest	5	0.9587	0.95	0.95	0.95

Acc, Accuracy; Pre, Precision; F-1, F-1 score; DWT, discrete wavelet transform; MAS, mean amplitude spectrum; CNN, convolutional neural network.

Currently, several studies have applied many frequency domain features for seizure detection and prediction and obtained valuable results. While PAC is more widely used for seizure onset detection and dynamic network connections in epilepsy, few studies have applied it to seizure prediction. Based on the experiments, the integration of PAC and machine learning may be a significant help to achieve an early warning of an imminent seizure for the adult database. Although the signal-channel PAC in our experiments is not absolutely predominant, our results show that the proposed method has the potential to become a reliable seizure detection and prediction tool for auxiliary clinical diagnosis and prediction. Moreover, the length of SPH is analyzed, and the results show that at a certain range, an SPH of 5 min has an overall performance on the seizure prediction. In clinical, it can be helpful to give timely aid before a seizure occurs. The next step will be to further explore the application of PAC in EEG data of children with epilepsy and to incorporate the algorithm into a practical EEG setting to support early intervention and hopefully improve the quality of life of patients.

## Data availability statement

The original contributions presented in the study are included in the article/Supplementary material, further inquiries can be directed to the corresponding author.

## Author contributions

XJ and XL contributed equally to this study. XJ, XL, YL, QW, BL, and LZ collected, processed, and analyzed epilepsy datasets to do seizure detection and prediction. All authors wrote and revised the study. All authors contributed to the article and approved the submitted version.
