# Supplementary material for: Epileptic seizures detection and the analysis of optimal seizure prediction horizon based on frequency and phase analysis
Source: Front Neurosci. 2023 May 16;17:1191683. doi: 10.3389/fnins.2023.1191683 (PMC10228742; doi:10.3389/fnins.2023.1191683)
Supplement: Supplementary file 1 [file Data_Sheet_1.docx]

Supplementary Material

**Epileptic Seizures Detection and the Analysis of Optimal Seizure Prediction Horizon based on Frequency-domain Analysis**

**Ximiao Jiang^1^, Xiaotong Liu^2#^, Youjun Liu^1^, Qingyun Wang^2^, Bao Li^1^, Liyuan Zhang^1*^**

*** Correspondence:**

Liyuan Zhang
[LiyuanZhang@bjut.edu.cn](mailto:LiyuanZhang@bjut.edu.cn)

# Supplementary Tables

**Supplementary Table 1** Results according to interval length in 12 patients from the Siena Scalp EEG database

| **Patient** | **5 minutes** | | | | | | | **10 minutes** | | | | | | | | **15 minutes** | | | | | | | |
| --- | --- | --- | --- | --- | --- | --- | --- | --- | --- | --- | --- | --- | --- | --- | --- | --- | --- | --- | --- | --- | --- | --- | --- |
|  | Acc | Pre | | Recall | | | F-1 | Acc | | Pre | | Recall | | | F-1 | Acc | | Pre | | Recall | | | F-1 |
| 1 | **87.12%** | | 0.87 | | 0.86 | 0.86 | | | **81.58%** | | 0.83 | | 0.80 | 0.80 | | | **79.53%** | | 0.81 | | 0.77 | 0.78 | |
| 3 | **94.66%** | | 0.96 | | 0.94 | 0.95 | | | **95.54%** | | 0.97 | | 0.95 | 0.96 | | | **95.59%** | | 0.97 | | 0.95 | 0.96 | |
| 5 | **81.47%** | | 0.84 | | 0.81 | 0.81 | | | **88.73%** | | 0.89 | | 0.88 | 0.88 | | | **88.22%** | | 0.89 | | 0.88 | 0.87 | |
| 6 | **86.31%** | | 0.86 | | 0.88 | 0.86 | | | **81.56%** | | 0.81 | | 0.78 | 0.78 | | | **81.84%** | | 0.82 | | 0.77 | 0.79 | |
| 7 | **94.44%** | | 0.96 | | 0.92 | 0.94 | | | **95.74%** | | 0.97 | | 0.94 | 0.96 | | | **94.96%** | | 0.96 | | 0.93 | 0.95 | |
| 9 | **79.87%** | | 0.79 | | 0.77 | 0.77 | | | **80.44%** | | 0.81 | | 0.80 | 0.80 | | | **81.09%** | | 0.82 | | 0.80 | 0.80 | |
| 11 | **87.01%** | | 0.87 | | 0.87 | 0.86 | | | **91.35%** | | 0.92 | | 0.91 | 0.91 | | | **91.93%** | | 0.93 | | 0.92 | 0.92 | |
| 12 | **80.69%** | | 0.82 | | 0.68 | 0.73 | | | **79.90%** | | 0.80 | | 0.68 | 0.73 | | | **78.47%** | | 0.80 | | 0.64 | 0.70 | |
| 13 | **77.05%** | | 0.79 | | 0.73 | 0.75 | | | **75.91%** | | 0.77 | | 0.71 | 0.73 | | | **74.76%** | | 0.76 | | 0.70 | 0.72 | |
| 14 | **91.59%** | | 0.92 | | 0.91 | 0.92 | | | **89.61%** | | 0.89 | | 0.91 | 0.89 | | | **89.77%** | | 0.89 | | 0.91 | 0.90 | |
| 16 | **79.60%** | | 0.80 | | 0.79 | 0.79 | | | **81.54%** | | 0.78 | | 0.89 | 0.82 | | | **81.63%** | | 0.78 | | 0.88 | 0.83 | |
| 17 | **88.68%** | | 0.92 | | 0.86 | 0.89 | | | **87.37%** | | 0.89 | | 0.85 | 0.87 | | | **86.78%** | | 0.89 | | 0.85 | 0.86 | |

Acc: Accuracy, Pre: Precision, F-1: F-1 score.

**Supplementary Table 2** Results according to interval length in 24 patients from the CHB-MIT dataset

| **Patient** | **5 minutes** | | | | | | | **10 minutes** | | | | | | | **15 minutes** | | | | | | |
| --- | --- | --- | --- | --- | --- | --- | --- | --- | --- | --- | --- | --- | --- | --- | --- | --- | --- | --- | --- | --- | --- |
|  | Acc | Pre | | Recall | | | F-1 | Acc | Pre | | Recall | | | F-1 | Acc | Pre | | Recall | | | F-1 |
| 1 | **98.47%** | | 0.98 | | 0.99 | 0.98 | | **98.95%** | | 0.98 | | 0.99 | 0.99 | | **98.87%** | | 0.99 | | 0.99 | 0.99 | |
| 2 | **98.90%** | | 0.99 | | 0.99 | 0.99 | | **98.98%** | | 0.99 | | 0.99 | 0.99 | | **98.66%** | | 0.99 | | 0.98 | 0.99 | |
| 3 | **92.93%** | | 0.93 | | 0.90 | 0.91 | | **92.68%** | | 0.93 | | 0.89 | 0.91 | | **93.05%** | | 0.93 | | 0.90 | 0.91 | |
| 4 | **94.77%** | | 0.92 | | 0.96 | 0.94 | | **94.10%** | | 0.90 | | 0.97 | 0.93 | | **94.47%** | | 0.92 | | 0.95 | 0.94 | |
| 5 | **98.42%** | | 0.98 | | 0.99 | 0.98 | | **98.82%** | | 0.99 | | 0.99 | 0.99 | | **98.85%** | | 0.99 | | 0.99 | 0.99 | |
| 6 | **97.41%** | | 0.97 | | 0.98 | 0.97 | | **96.81%** | | 0.96 | | 0.99 | 0.98 | | **97.30%** | | 0.96 | | 1.10 | 0.97 | |
| 7 | **99.09%** | | 1.00 | | 0.99 | 0.99 | | **98.11%** | | 0.99 | | 0.97 | 0.98 | | **97.93%** | | 0.98 | | 0.97 | 0.98 | |
| 8 | **95.32%** | | 0.97 | | 0.94 | 0.95 | | **95.72%** | | 0.96 | | 0.96 | 0.96 | | **93.23%** | | 0.93 | | 0.94 | 0.94 | |
| 9 | **98.96%** | | 0.99 | | 0.99 | 0.99 | | **98.58%** | | 0.99 | | 0.99 | 0.99 | | **98.23%** | | 0.99 | | 0.98 | 0.98 | |
| 10 | **96.71%** | | 0.96 | | 0.97 | 0.96 | | **96.03%** | | 0.97 | | 0.93 | 0.95 | | **95.14%** | | 0.96 | | 0.92 | 0.94 | |
| 11 | **91.02%** | | 0.88 | | 0.74 | 0.80 | | **91.15%** | | 0.91 | | 0.72 | 0.80 | | **-** | | - | | - | - | |
| 12 | **91.90%** | | 0.91 | | 0.92 | 0.91 | | **92.43%** | | 0.90 | | 0.96 | 0.93 | | **90.70%** | | 0.89 | | 0.91 | 0.90 | |
| 13 | **97.62%** | | 0.97 | | 0.97 | 0.97 | | **97.17%** | | 0.97 | | 0.95 | 0.96 | | **97.79%** | | 0.99 | | 0.94 | 0.96 | |
| 14 | **96.04%** | | 0.96 | | 0.95 | 0.95 | | **95.75%** | | 0.94 | | 0.95 | 0.95 | | **96.99%** | | 0.97 | | 0.96 | 0.96 | |
| 15 | **94.62%** | | 0.94 | | 0.94 | 0.94 | | **95.88%** | | 0.96 | | 0.95 | 0.95 | | **96.24%** | | 0.96 | | 0.95 | 0.95 | |
| 16 | **94.94%** | | 0.97 | | 0.93 | 0.95 | | **91.90%** | | 0.94 | | 0.89 | 0.92 | | **96.63%** | | 0.97 | | 0.96 | 0.97 | |
| 17 | **97.73%** | | 0.98 | | 0.98 | 0.98 | | **98.55%** | | 0.99 | | 0.99 | 0.99 | | **97.93%** | | 0.99 | | 0.98 | 0.98 | |
| 18 | **92.48%** | | 0.85 | | 0.90 | 0.88 | | **92.56%** | | 0.95 | | 0.59 | 0.72 | | **92.57%** | | 0.90 | | 0.63 | 0.74 | |
| 19 | **99.17%** | | 0.99 | | 0.99 | 0.99 | | **99.39%** | | 1.00 | | 1.00 | 0.99 | | **98.68%** | | 0.99 | | 0.99 | 0.99 | |
| 20 | **95.69%** | | 0.94 | | 0.97 | 0.95 | | **95.67%** | | 0.93 | | 0.98 | 0.95 | | **95.39%** | | 0.94 | | 0.97 | 0.95 | |
| 21 | **93.63%** | | 0.94 | | 0.91 | 0.92 | | **94.40%** | | 0.95 | | 0.92 | 0.93 | | **94.71%** | | 0.94 | | 0.93 | 0.94 | |
| 22 | **92.98%** | | 0.94 | | 0.89 | 0.91 | | **92.55%** | | 0.94 | | 0.87 | 0.90 | | **92.92%** | | 0.95 | | 0.90 | 0.92 | |
| 23 | **97.50%** | | 0.97 | | 0.98 | 0.98 | | **96.47%** | | 0.97 | | 0.96 | 0.96 | | **97.17%** | | 0.97 | | 0.97 | 0.97 | |
| 24 | **94.51%** | | 0.93 | | 0.97 | 0.95 | | **92.28%** | | 0.91 | | 0.94 | 0.92 | | **93.97%** | | 0.92 | | 0.95 | 0.93 | |

Acc: Accuracy, Pre: Precision, F-1: F-1 score.

# Supplementary Figures


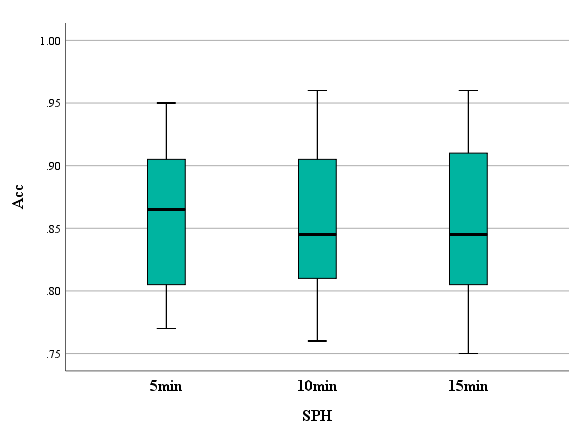


**Supplementary Figure 1** Kruskal-Wallis test between 5min, 10min and 15min SPH from the Siena Scalp EEG database. There was no significant difference (p>0.05).


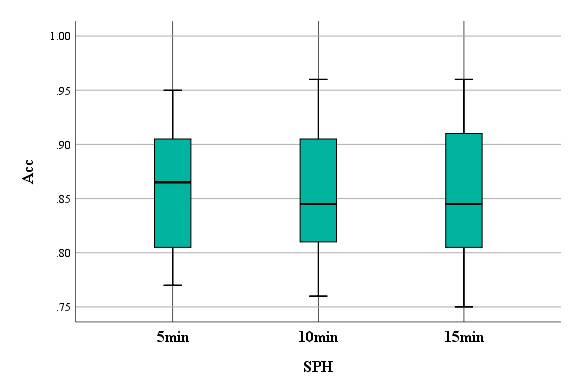


**Supplementary Figure 2** Kruskal-Wallis test between 5min, 10min and 15min SPH from the CHB-MIT dataset. There was no significant difference (p>0.05).
